# Protective Effect of *Castanopsis sieboldii* Extract against UVB-Induced Photodamage in Keratinocytes

**DOI:** 10.3390/molecules28062842

**Published:** 2023-03-21

**Authors:** Hye Rim Lee, Ji Hye Yang, Ji Hyun Lee, Kyu Min Kim, Sam Seok Cho, Jin Sol Baek, Jae Min Kim, Moon-Hee Choi, Hyun-Jae Shin, Sung Hwan Ki

**Affiliations:** 1 College of Pharmacy, Chosun University, Gwangju 61452, Republic of Korea; 2 College of Korean Medicine, Dongshin University, Naju 58245, Republic of Korea; 3 Department of Biomedical Science, College of Natural Science, Chosun University, Gwangju 61452, Republic of Korea; 4 Department of Biochemical Engineering, College of Engineering, Chosun University, Gwangju 61452, Republic of Korea

**Keywords:** *Castanopsis sieboldii*, UVB, keratinocyte, autophagy, ER stress

## Abstract

Ultraviolet B (UVB) rays disrupt the skin by causing photodamage via processes such as reactive oxygen species (ROS) production, endoplasmic reticulum (ER) stress, DNA damage, and/or collagen degradation. *Castanopsis sieboldii* is an evergreen tree native to the southern Korean peninsula. Although it is known to have antioxidant and anti-inflammatory effects, its protective effect against photodamage in keratinocytes has not been investigated. Thus, in the present study, we investigated the effect of 70% ethanol extract of *C. sieboldii* leaf (CSL3) on UVB-mediated skin injuries and elucidated the underlying molecular mechanisms. CSL3 treatment restored the cell viability decreased by UVB irradiation. Moreover, CSL3 significantly inhibited UVB- or tert-butyl hydroperoxide-mediated ROS generation in HaCaT cells. ER stress was inhibited, whereas autophagy was upregulated by CSL3 treatment against UVB irradiation. Additionally, CSL3 increased collagen accumulation and cell migration, which were decreased by UVB exposure. Notably, epigallocatechin gallate, the major component of CSL3, improved the cell viability decreased by UVB irradiation through regulation of ER stress and autophagy. Conclusively, CSL3 may represent a promising therapeutic candidate for the treatment of UVB-induced skin damage.

## 1. Introduction

The skin is the largest organ in the body and comprises three layers: the epidermis, dermis, and subcutaneous tissue. Skin aging is a process in which intrinsic and extrinsic determinants progressively lead to a loss of structural integrity and physiological function [1]. Intrinsic skin aging occurs inevitably as a natural consequence of physiological changes over time at variable yet inalterable genetically determined rates. Extrinsic factors are controllable and include exposure to ultraviolet (UV) rays, pollution or nicotine, repetitive muscle movements, and miscellaneous lifestyle components such as diet, sleeping position, and overall health [2].

Among them, UV exposure is a major causative factor for age-associated changes, including oxidative stress, inflammation, degradation of the extracellular matrix (ECM), and skin aging. The skin is readily exposed to UV radiation, which is composed of UVA (315–400 nm), UVB (280–315 nm), and UVC (100–280 nm) [3]. UVC is absorbed by the ozone layer, but UVA and UVB can penetrate the skin. Importantly, UVB has a longer wavelength than UVA and causes greater skin damage. Ultraviolet (UV) induces cell damage by causing DNA damage, such as cyclobutane pyrimidine dimer [4,5]. Furthermore, UVB generates reactive oxygen species (ROS) production, which eventually leads to various skin disorders, including inflammation, aging, and cancer [6]. Additionally, the ROS produced can induce programmed cell death via apoptosis and autophagy or unprogrammed cell death via necrosis [7]. Thus, induced ROS production by UVB is responsible for skin damage, and substance with antioxidant capacity is expected to protect against skin damage by UVB.

Skin aging is caused by UVB irradiation-induced photodamage and is characterized by degradation of the ECM [8,9]. The ECM plays an essential role in cell adhesion, cell-to-cell communication, and differentiation of skin cells. UVB degrades ECM components, such as collagen and elastin, by inducing matrix metalloproteinase (MMP) expression. Specifically, MMP3 and MMP9 act as major collagenases [10]. MMP expression is transcriptionally regulated by activator protein 1 (AP-1) and is upregulated by UVB-induced c-Jun or c-Fos [11,12]. Therefore, MMP upregulation by UVB may cause dermal aging. The epidermis predominantly comprises keratinocytes, representing >95% of the total cell population [13]. Keratinocytes protect the skin from external factors that induce aging [14]. However, UVB induces photodamage and skin aging by activating MMP [15].

Endoplasmic reticulum (ER) stress is generated by misfolded and unfolded proteins through unfolded protein response (UPR). Repeated ER stress is eventually followed by cell death [16]. Therefore, removing damaged ER is important for maintaining cellular homeostasis. Autophagy is an essential lysosome-dependent cell survival mechanism that involves the degradation and recycling of unfolded proteins and intracellular components through various stimuli [17]. The mammalian target of rapamycin (mTOR), a serine/threonine kinase, is a master regulator of cellular metabolism and a sensor of cellular nutritional status. The mTOR signaling pathway plays a crucial role in autophagy regulation, and inhibition of mTOR potently induces autophagy [18]. Recently, activation of mTOR has been reported to increase damaged ER and mitochondria [19]. Hence, mTOR inhibition may be an effective strategy for reducing ER stress and its related diseases. Mitogen-activated protein kinases (MAPKs) are the major upstream modulators of mTOR and can reduce ER stress [20].

*Castanopsis sieboldii* (CS) is an evergreen tree species in subtropical eastern Asia, including Korea. It is known to have antioxidant activity and reduce oxidative stress by decreasing intracellular ROS levels. In addition, CS has high 2,2-diphenyl-1-picrylhydrazyl (DPPH) and 2,2-azino-bis (3-ethylbenzthiazoline-6-sulfonic acid) diammonium salt (ABTS) radical scavenging activities [21]. Its radical scavenging capability prevents lipid peroxidation, cellular DNA damage, and, consequently, apoptosis in lung fibroblasts V79-4 cells treated with H_2_O_2_ [22]. Moreover, CS can inhibit inflammatory responses in macrophages via LPS stimulation [23]. Although CS has been known to exert a number of pharmacological actions, such as the anti-oxidative and anti-inflammatory effects mentioned above, the pharmacological efficacy of CS against skin damage in UVB-irradiated keratinocytes remains unexplored.

In the present study, we evaluated the cytoprotective effect of 70% ethanol (EtOH) extract of *C*S leaf (CSL) on HaCaT cells (human keratinocyte cell line) irradiated with UVB. Our results implied that UVB increases ROS generation and cytotoxicity, which are attenuated by treatment with 70% EtOH CSL (CSL3) extract via autophagy-dependent ER stress inhibition. Furthermore, quantitative analysis of CSL3 was performed using high-performance liquid chromatography-mass spectrometry (HPLC–MS), and the composition of polyphenols was investigated. Therefore, CSL3 may represent a promising and effective agent against UVB irradiation-mediated skin damage.

## 2. Results

### 2.1. CSL3 Has Higher Anti-oxidative Efficacy Compared with Other CSL Extracts

**Figure 1:**
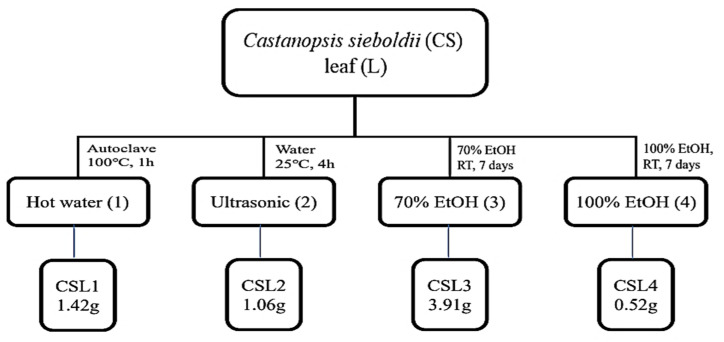
Schematic diagram of extraction methods of *Castanopsis sieboldii* leaves.

**Figure 2:**
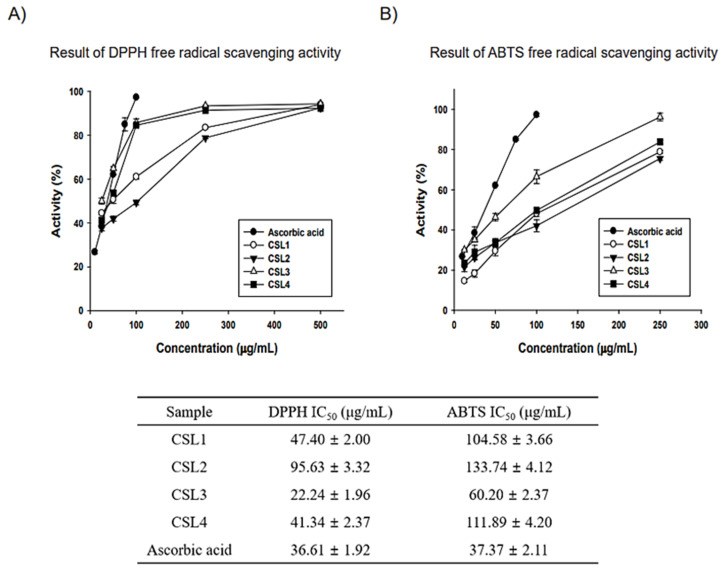
Antioxidative efficacy measurements of CSL extracts. (**A**,**B**) Antioxidant activity results: 2,2-diphenyl-1-picrylhydrazyl (DPPH) and 2,2-azino-bis (3-ethylbenzthiazoline-6-sulfonic acid) diammonium salt (ABTS). Experiments were performed in triplicate and repeated three times with similar results.

CSL was extracted using water, an ultrasonic bath, and ethanol (70% or 100%) (Figure 1). The antioxidant properties of CSL extracts were assayed for radical scavenging activities using DPPH and ABTS (Figure 2). Ascorbic acid was used as a positive control, and the vehicle did not affect IC_50_. The extract concentrations ranged from 10–500 μg/mL, and the IC_50_ of CSL3 was 22.24 ± 1.96 μg/mL and 60.20 ± 2.37 μg/mL in the DPPH and ABTS assays, respectively. CSL3 had the highest antioxidant activity among the extracts in all radical scavenging activity assays. Ascorbic acid, used as a positive control, exhibited an IC_50_ of 36.61 ± 1.92 μg/mL in the DPPH assay and 37.37 ± 2.11 μg/mL in the ABTS assay. In addition, we recently identified the antioxidant capacity of five fractions of CSL3 (n-hexane, chloroform, ethyl acetate, n-butanol, and water) and isolated polyphenolic compounds [24]. Experimental results revealed that EtOAc fractions of CSL3 had the highest radical scavenging activity and contained total polyphenol contents (TPC) and total flavonoid contents (TFC). In a previous study, the IC_50_ value of 80% ethanol extracts of CSL was 16.26 μg/mL [25], similar to that of the present study. Youn et al. measured the antioxidant activities of a methanol extract of the nuts of CS at a concentration of 50 μg/mL [21]. DPPH and ABTS activities were 75% and 98%, respectively, with very high antioxidant activities at low concentrations. In conclusion, CSL3 had higher antioxidative efficacy compared with the other CSL extracts.

### 2.2. Composition of CSL Extracts

**Figure 3:**
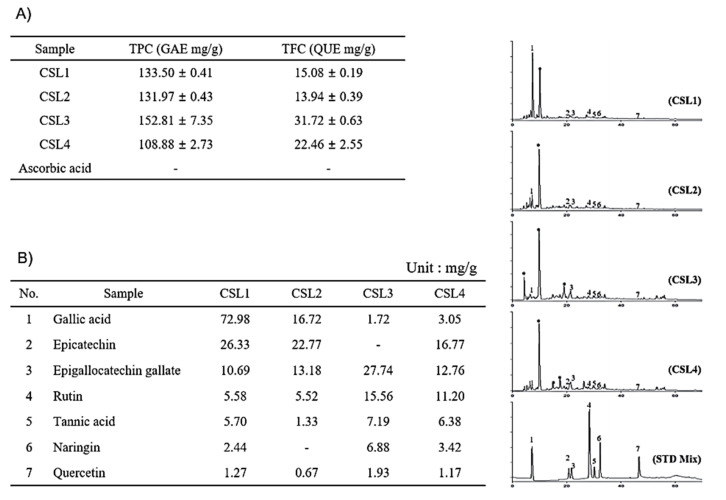
Identification of components in CSL3 and CSL extracts. (**A**) The TPC and TFC were determined using colorimetric methods. (**B**) HPLC chromatogram of CSL extracts. Gallic acid (peak 1), epicatechin (peak 2), EGCG (peak 3), rutin (peak 4), tannic acid (peak 5), naringin (peak 6), and quercetin (peak 7).

Thereafter, we analyzed the composition of CSL extracts. The TPC and TFC were determined using colorimetric methods. Gallic acid and quercetin were used as equivalent substances. CSL3 had the highest content of phenolic compounds (Figure 3A). The TPC and TFC of CSL3 were 152.81 ± 7.35 gallic acid equivalent mg/g and 31.72 ± 0.63 quercetin equivalent mg/g, respectively. Similar to the antioxidant activity results, CSL3 had a higher TPC than CSL4, indicating that CSL3 contained high-polarity antioxidants. Additionally, as flavonoids have relatively low polarity, it was confirmed that TFC was increased during organic solvent extraction (CSL3 and CSL4).

To identify the components in CSL3 and CSL extracts, we chose seven phenolic acid and flavonoid standards (gallic acid, epicatechin, epigallocatechin gallate, rutin, tannic acid, naringin, and quercetin) and performed HPLC. We found that the major component of CSL3 was epigallocatechin gallate (EGCG), a well-known antioxidant polyphenol effective against hydrogen peroxide and radical-scavenging activity [26,27] (Figure 3B).

LC-MS/MS analysis was performed to confirm HPLC results. We identified 15 polyphenols and quantitatively analyzed them in CSL1-4 extract by LC-MS/MS system. Among them, CSL3 had the highest content of polyphenols with 4264.20 ug/g, followed by CSL1, CSL2, and CSL4 (Figure S1). In our previous study, the components detected in the EtOAc fraction of the CS were high in the order of ethyl gallate, gallic acid, and chlorogenic acid [24]. However, the content of EGCG was the highest, and gallic acid, protocatechuic acid, and chlorogenic acid were mainly detected in the current study. Therefore, it is thought that the content and components detected depend on the extraction condition of the CS.

### 2.3. CSL3 Protects HaCaT Cells by Reducing Apoptosis

**Figure 4:**
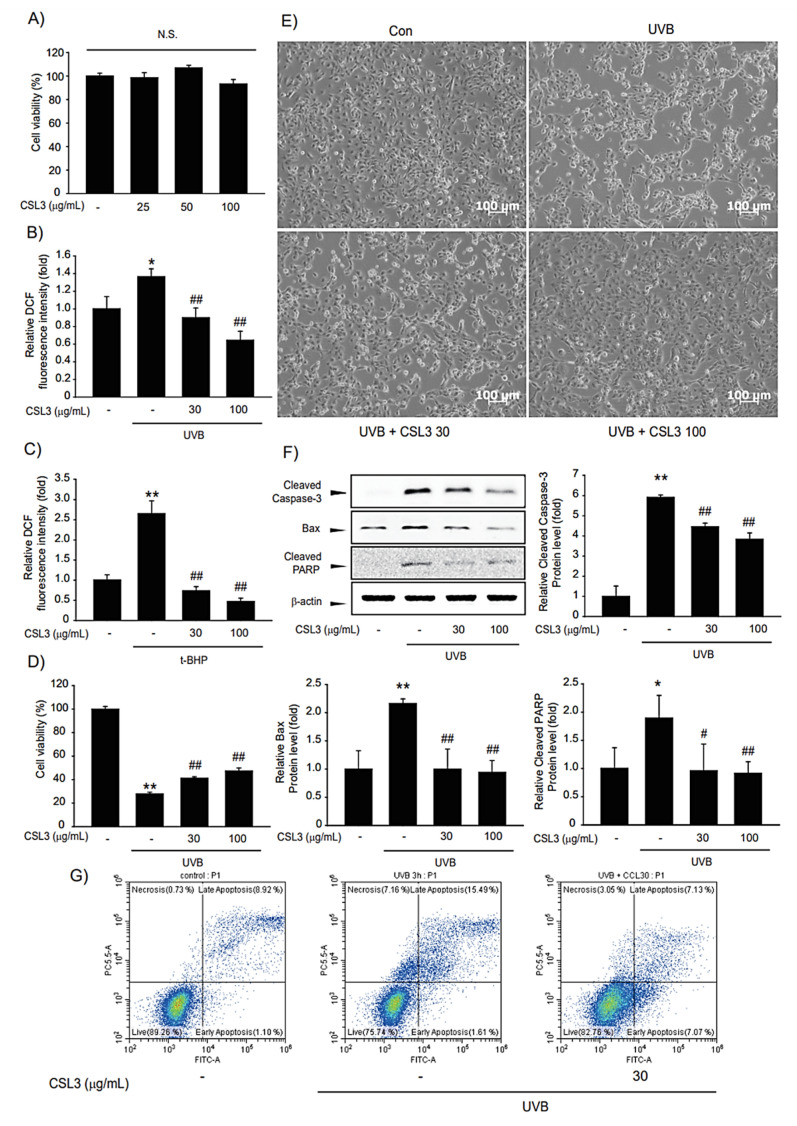
Effects of CSL3 on the viability of HaCaT cells. (**A**) The cytotoxic effect of CSL3 in HaCaT cells was determined by MTT assays. Cells were treated with CSL3 (25–100 μg/mL) for 12 h. Data represent means ± S.E. of 3 replicates; significant when compared with respective controls, N.S.—not significant. (**B**,**C**) The effect of CSL3 on intercellular ROS production induced by UVB irradiation (30 mJ/cm^2^) or *t*-BHP treatment (600 μM) in HaCaT cells. First, we pre-treated with CSL3 (30–100 μg/mL) for 1 h in HaCaT cells. Then, the cells were exposed to UVB irradiation or *t*-BHP treatment, followed by incubation with CSL3 (30–100 μg/mL) for 1 h. Data represent means ± S.E. of 3 replicates; significant when compared with respective controls, *p < 0.05, ** *p* < 0.01, compared with the UVB irradiation or *t*-BHP treatment group, ^##^ *p* < 0.01. (**D**) Cytoprotective effects of CSL3 against UVB irradiation in HaCaT cells. We first pre-treated with CSL3 (30–100 μg/mL) for 1 h in HaCaT cells. Then, the cells were exposed to UVB irradiation (30 mJ/cm^2^), followed by incubation with CSL3 (30–100 μg/mL) for 6 h. Cell viability was assessed via MTT assays. Data represent means ± S.E. of 3 replicates; significant when compared with respective controls, ** *p* < 0.01, compared with the UVB irradiation group, ^#^ *p* < 0.05, ^##^ *p* < 0.01. (**E**) Microscopic analysis. HaCaT cells were pretreated with CSL3 (30–100 μg/mL) for 1 h. Cells were then irradiated with UVB irradiation (30 mJ/cm^2^), followed by incubation with CSL3 for 3 h. Cell morphology was visualized using a microscope (magnification: 100×). (**F**) Effects of CSL3 on apoptosis. HaCaT cells were pretreated with CSL3 (30–100 μg/mL) for 1 h. Cells were then irradiated with UVB irradiation (30 mJ/cm^2^), followed by incubation with CSL3 for 1 h. Proteins associated with apoptosis were examined via immunoblotting. Data represent means ± S.E. of 3 replicates; significant when compared with respective controls, ** *p* < 0.01, compared with the UVB irradiation groups, ^##^ *p* < 0.01. (**G**) Flow cytometry analysis of apoptosis. HaCaT cells were pretreated with CSL3 for 1 h. Cells were then irradiated with UVB irradiation (30 mJ/cm^2^), followed by incubation with CSL3 (30–100 μg/mL) for 1 h. Apoptosis levels were measured via flow cytometry. Results were confirmed by repeated 3 experiments.

To check the cytotoxicity of CSL3 in keratinocytes, we performed MTT assays with various CSL3 concentrations (25, 50, and 100 μg/mL) for 12 h in HaCaT cells. Treatment of HaCaT cells with up to 100 μg/mL of CSL3 did not exhibit a significantly different result from that of the vehicle-only treated group (Figure 4A). Thereafter, we evaluated the anti-oxidative capacity of CSL3 by using DCFH-DA dye. CSL3 considerably inhibited the increased ROS generation induced by UVB (312 nm) irradiation (30 mJ/cm^2^) (Figure 4B). Moreover, treatment with CSL3 antagonized ROS production by *t*-BHP (600 μM) (Figure 4C) in HaCaT cells. Furthermore, we assessed the cytoprotective effect of CSL3 against UVB irradiation using MTT assays in HaCaT cells. We discovered that CSL3 improved the decreased cell viability caused by UVB irradiation in a concentration-dependent manner (Figure 4D). Microscopic analysis revealed that CSL3 improved the viability of UVB-damaged HaCaT cells (Figure 4E). UVB is known to cause apoptosis [28]. Thus, we evaluated whether CSL3 can inhibit apoptosis induced by UVB irradiation. UVB-induced increase in caspase-3 cleavage was effectively attenuated by CSL3 pretreatment. Expression of the apoptosis regulators Bax and PARP was also decreased by CSL3 treatment (Figure 4F). Additionally, apoptosis levels were examined using Annexin V/PI staining, and the results showed that CSL3 treatment alleviated the increased apoptosis caused by UVB irradiation (Figure 4G). Collectively, these results imply that the antioxidant capacity of CSL3 inhibits UVB-induced HaCaT cell apoptosis.

### 2.4. CSL3 Inhibits ER Stress Caused by UVB Irradiation

**Figure 5:**
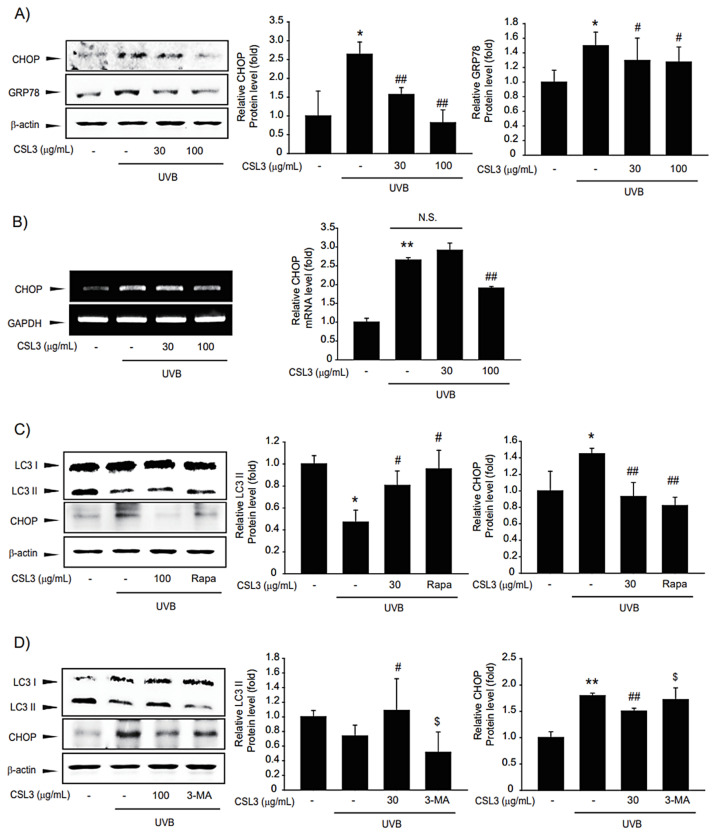
Effects of CSL3 on ER stress in HaCaT cells irradiated with UVB. (**A**) Effects of CSL3 on UVB-induced ER stress. First, we pre-treated with CSL3 (30–100 μg/mL) for 1 h in HaCaT cells. Then, the cells were exposed to UVB irradiation (30 mJ/cm^2^), followed by incubation with CSL3 for 3 h. ER stress marker proteins were visualized using immunoblotting. Data represent means ± S.E. of 3 replicates; significant when compared with respective controls, * *p* < 0.05, compared with the UVB irradiation group, ^#^ *p* < 0.05, ^##^ *p* < 0.01. (**B**) RT-PCR analysis. We first pre-treated with CSL3 (30–100 μg/mL) for 1 h in HaCaT cells. Then, the cells were exposed to UVB irradiation (30 mJ/cm^2^), followed by incubation with CSL3 for 3 h. Data represent means ± S.E. of 3 replicates; significant when compared with respective controls, ** *p* < 0.01, compared with the UVB irradiation group, ^##^ *p* < 0.01. N.S.—not significant. (**C**) We first pre-treated with CSL3 (100 μg/mL) or rapamycin (500 nM) for 1 h in HaCaT cells. Then, the cells were exposed to UVB irradiation (30 mJ/cm^2^), followed by incubation with CSL3 or rapamycin for 3 h. Expressions of LC3II and CHOP proteins were visualized using immunoblotting. Data represent means ± S.E. of 3 replicates; significant when compared with respective controls, * *p* < 0.05, compared with the UVB irradiation group, ^#^ *p* < 0.05, ^##^ *p* < 0.01. (**D**) HaCaT cells were pretreated with CSL3 (100 μg/mL) or 3-MA (50 μM) for 1 h. Cells were then irradiated with UVB irradiation (30 mJ/cm^2^), followed by incubation with CSL3 or 3-MA for 3h. Expressions of LC3II and CHOP proteins were visualized using immunoblotting. Data represent means ± S.E. of 3 replicates; significant when compared with respective controls, ** *p* < 0.01, compared with the UVB irradiation group, ^#^ *p* < 0.05, ^##^ *p* < 0.01, compared with UVB irradiation and CSL3 treatment ^$^ *p* < 0.05.

It is well-established that UVB-induced apoptosis is regulated by ER stress [29]. Thus, we investigated the protective capacity of CSL3 on UVB-induced ER stress. Immunoblotting data showed that the expression of CHOP and GRP78, representative ER stress markers, was increased by UVB irradiation, whereas CSL3 inhibited the expression of these markers (Figure 5A). Furthermore, RT-PCR analysis confirmed that CSL3 inhibited UVB-induced ER stress (Figure 5B).

Recently, it was reported that UVB-induced ER stress is modulated by autophagy, which is crucial for maintaining cellular homeostasis in keratinocytes [14]. In addition, autophagy induction can repair cell organelles damaged by various stresses [30]. UVB irradiation inhibited autophagy, as evidenced by reduced expression of LC3B-II, which was reversed by treatment with CSL3. Rapamycin was used as a positive control (Figure 5C), and CSL3 alone did not affect LC3II and CHOP expression (Figure S2). 3-Methyladenine, a representative autophagy inhibitor, antagonized the inhibition of UVB-mediated CHOP expression by CSL3 (Figure 5D). Collectively, our data showed that CSL3 alleviated UVB-induced ER stress via autophagy induction.

### 2.5. CSL3 Induces Autophagy through mTOR Pathway

**Figure 6:**
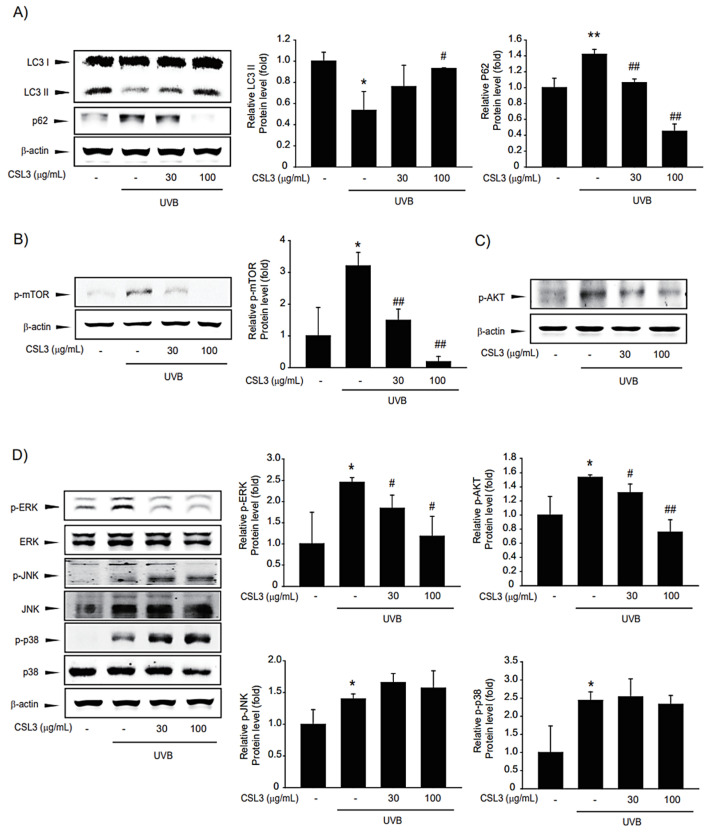
Effects of CSL3 on autophagy in UVB-irradiated HaCaT cells. (**A**) Effect of CSL3 on autophagy. First, we pre-treated with CSL3 (30–100 μg/mL) for 1 h in HaCaT cells. Then, the cells were exposed to UVB irradiation (30 mJ/cm^2^), followed by incubation with CSL3 for 3 h. LC3B and p62 expression were visualized using immunoblotting. Data represent means ± S.E. of 3 replicates; significant when compared with respective controls, * *p* < 0.05, ** *p* < 0.01, compared with the UVB irradiation group, ^#^ *p* < 0.05, ^##^ *p* < 0.01. (**B**–**D**) We first pre-treated with CSL3 (30–100 μg/mL) for 1 h in HaCaT cells. Then, the cells were exposed to UVB irradiation (30 mJ/cm^2^), followed by incubation with CSL3 for 3 h. Expressions of p-Akt, p-mTOR, and MAPKs (p-ERK, ERK, p-JNK, JNK, p-p38, and p38) proteins were measured using immunoblotting. Data represent means ± S.E. of 3 replicates; significant when compared with respective controls, * *p* < 0.05, compared with the UVB irradiation group, ^#^ *p* < 0.05, ^##^ *p* < 0.01.

Autophagy is an important pathway for regulating cell homeostasis [17,31]. UVB reduced the expression of LC3B-II, a representative autophagy induction marker; however, CSL3 reversed this phenomenon (Figure 6A). Furthermore, we examined whether CSL3 induced autophagy via the mTOR pathway. We found that UVB-induced mTOR phosphorylation was decreased by CSL3 (Figure 6B). Additionally, CSL3 antagonized phosphorylation of Akt, a well-known regulator of mTOR activity (Figure 6C). We then evaluated the MAPK signaling pathway, which is another upstream pathway of mTOR [32]. CSL3 considerably inhibited ERK phosphorylation but not JNK and p38 phosphorylation (Figure 6D). These results demonstrated that CSL3 could protect UVB-damaged HaCaT cells by autophagy induction through the mTOR signaling pathway.

### 2.6. CSL3 Decreases MMP Production by UVB

**Figure 7:**
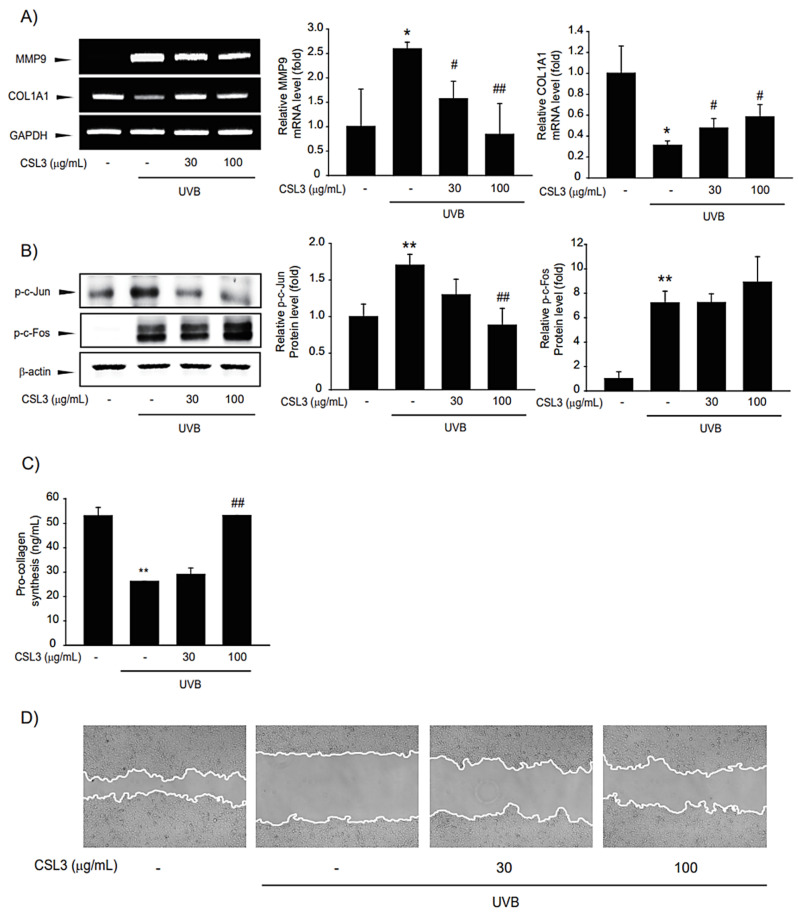
Effect of CSL3 on MMP9 and collagen expression in HaCaT cells. (**A**) First, we pre-treated with CSL3 (30–100 μg/mL) for 1 h in HaCaT cells. Then, the cells were exposed to UVB irradiation (30 mJ/cm^2^), followed by incubation with CSL3 for 3 h. mRNA levels of *MMP9* and *collagen* were evaluated using RT-PCR analysis. Data represent means ± S.E. of 3 replicates; significant when compared with respective controls, * *p* < 0.05, compared with the UVB irradiation group, ^#^ *p* < 0.05, ^##^ *p* < 0.01. (**B**) We first pre-treated with CSL3 (30–100 μg/mL) for 1 h in HaCaT cells. Then, the cells were exposed to UVB irradiation (30 mJ/cm^2^), followed by incubation with CSL3 for 3 h. Protein expression of AP-1 was visualized using immunoblotting. Data represent means ± S.E. of 3 replicates; significant when compared with respective controls, ** *p* < 0.01, compared with the UVB irradiation group, ^##^ *p* < 0.01. (**C**) HaCaT cells were pretreated with CSL3 (30–100 μg/mL) for 1 h. Cells were then irradiated with UVB irradiation (30 mJ/cm^2^), followed by incubation with CSL3 for 6 h. Pro-collagen expression was measured by using Pro-collagen Type I C-peptide EIA Kit. Data represent means ± S.E. of 3 replicates; significant when compared with respective controls, ** *p* < 0.01, compared with the UVB irradiation group, ^##^ *p* < 0.01. (**D**) Wound-healing assay. We first pre-treated with CSL3 (30–100 μg/mL) for 1 h in HaCaT cells. Then, the cells were exposed to UVB irradiation (30 mJ/cm^2^), followed by incubation with CSL3 for 12 h. The results were visualized via microscopy after UVB irradiation. Results were confirmed by repeated 3 experiments.

ECM degradation and increased MMPs are characteristic of skin aging. UVB inhibits collagen synthesis by increasing MMP activity. Therefore, we evaluated the anti-dermal aging effect of CSL3. Our results showed that UVB inhibited collagen accumulation but enhanced MMP9 expression (Figure 7A). AP-1 is composed of c-Jun and c-Fos and is known to regulate MMP expression [33]. Hence, we evaluated the phosphorylation levels of c-Jun and c-Fos. Increased c-Jun phosphorylation was suppressed by pretreatment with CSL3; however, c-Fos phosphorylation was not affected by CSL3 treatment (Figure 7B). Therefore, we concluded that CSL3 regulates the expression of MMP through the regulation of c-Jun rather than c-Fos. Thereafter, we analyzed collagen accumulation and confirmed that UVB decreased collagen levels; this phenomenon was inhibited by CSL3 pretreatment (Figure 7C). Furthermore, we evaluated the migration of HaCaT cells treated with CSL3 using a wound-healing assay. CSL3 inhibited the decreased cell migration caused by UVB irradiation (Figure 7D). These results indicated that CSL3 could decrease MMP expression and increase collagen accumulation.

### 2.7. Cytoprotective Effect of EGCG in UVB-Damaged HaCaT Cells

**Figure 8:**
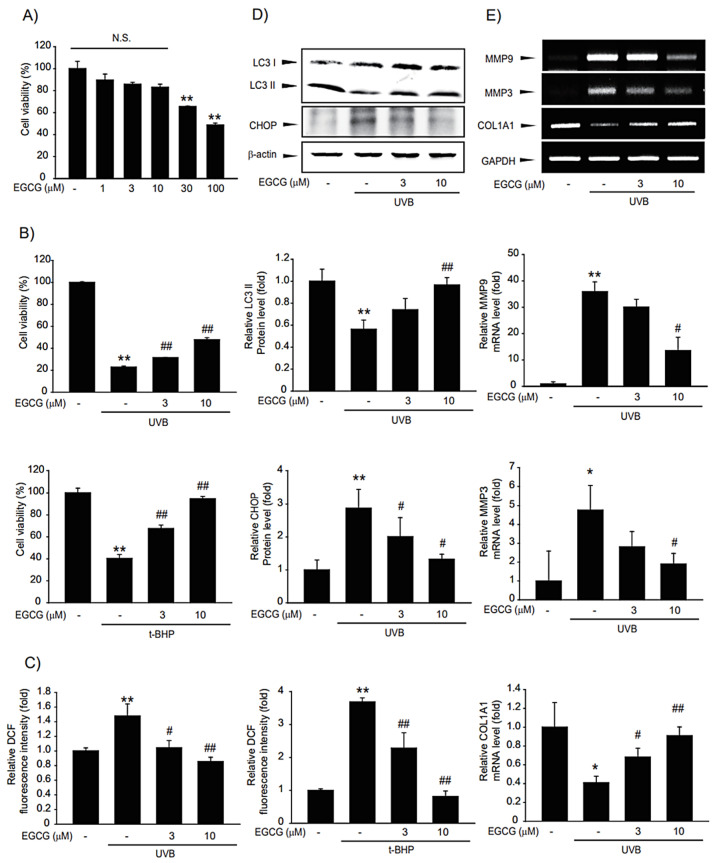
Effects of epigallocatechin (EGCG), the major component of CSL3, on UVB-photodamaged HaCaT cells. (**A**) Cytotoxic effects of EGCG in HaCaT cells were determined by MTT assays. Cells were treated with EGCG (1–100 μM) for 12 h. Data represent means ± S.E. of 3 replicates; significant when compared with respective controls, ** *p* < 0.01. N.S., not significant (**B**) The cytoprotective effects of EGCG on photodamaged HaCaT cells were evaluated by MTT assays. We first pre-treated with EGCG (3–10 μM) for 1 h in HaCaT cells. Then, the cells were exposed to UVB irradiation (30 mJ/cm^2^) or *t*-BHP treatment (600 μM), followed by incubation with EGCG for 6 h. Cell viability was measured using MTT assay. Data represent means ± S.E. of three replicates; significant when compared with controls, * *p* < 0.05, ** *p* < 0.01, compared with the UVB irradiation or *t*-BHP treatment groups, ^#^ *p* < 0.05, ^##^ *p* < 0.01. (**C**) Effects of EGCG on intercellular ROS production. We first pre-treated with EGCG (3–10 μM) for 1 h in HaCaT cells. Then, the cells were exposed to UVB irradiation (30 mJ/cm^2^) or *t*-BHP (600 μM) treatment, followed by incubation with EGCG for 1 h. Results were confirmed by repeated 3 experiments. (**D**) HaCaT cells were pretreated with EGCG (3–10 μM) for 1 h. Then the cells were irradiated with UVB irradiation (30 mJ/cm^2^), followed by incubation with EGCG for 3 h. Expressions of autophagy markers (LC3B and p62) and ER stress markers (CHOP, GRP78) were measured using immunoblotting. Results were confirmed by repeated 3 experiments. (**E**) RT-PCR analysis. We first pre-treated with EGCG (3–10 μM) for 1 h in HaCaT cells. Then, the cells were exposed to UVB irradiation (30 mJ/cm^2^), followed by incubation with EGCG for 3 h.

We evaluated whether the CSL3 effect was due to EGCG, a major component of CSL3. First, we tested the cytotoxic effect of EGCG using MTT assays. Concentrations ranging from 1–10 μM were not cytotoxic, but higher concentrations exerted toxicity (Figure 8A). Thus, we used up to 10 μM of EGCG for studying cytoprotective effects. EGCG efficiently improved the cell viability impacted by UVB irradiation or *t*-BHP treatment (Figure 8B). We further evaluated whether EGCG could decrease UVB-induced ROS production. EGCG reduced intracellular ROS levels induced by UVB or *t*-BHP (Figure 8C). We inferred that CSL3 protects UVB-damaged HaCaT cells through autophagy induction and ER stress inhibition. Consistent with CSL3, EGCG reversed UVB-mediated autophagy inhibition and decreased ER stress (Figure 8D). Finally, we investigated the protective effect of EGCG against skin damage. EGCG decreased MMP expression but enhanced collagen expression (Figure 8E). Therefore, we concluded that the cytoprotective effects of CSL3 in HaCaT cells against UVB-mediated damage were due to EGCG.

## 3. Discussion

Protecting skin cells is crucial because the skin acts as the primary defense organ of our body and is always exposed to external stimuli. The skin is easily exposed to UVB in the external environment, which is a major cause of dermal diseases [34,35]. A UVB comprises light-emitting diodes (LEDs) and mercury vapor lamps. Recently, research on UVB LEDs has been conducted because LEDs are more eco-friendly than mercury vapor UVB lamps and use less energy. However, mercury vapor UVB lamps are still used in many industrial, agricultural, and medical fields for reasons of high efficiency [36,37]. We used mercury vapor UVB lamps as the light source to induce damage in keratinocytes. UVB lamps increase ROS formation, resulting in oxidative stress and various insults, such as DNA damage and cell death, in the skin. Here, we found that CSL3 protected keratinocytes against UVB-induced photodamage by suppressing ER stress and autophagy activation.

Moreover, we examined whether CSL3 can absorb UVB rays. As a result, the absorbance value at 312 nm was increased in a concentration-dependent manner by CSL3 but not DMSO (Figure S3). These results indicated that the CSL3 effect was partially due to its UVB-absorbing capability. In addition, CSL3 had higher anti-oxidative efficacy compared with other CSL extracts (Figure 2). Recently we demonstrated the antioxidant capacity of five fractions of CSL3 (n-hexane, chloroform, ethyl acetate, n-butanol, and water) and isolated polyphenolic compounds [38]. Experimental results revealed that EtOAc fractions of CSL3 exhibited the highest radical scavenging activity and contained TPC and TFC. Furthermore, we identified that CSL3 significantly decreased ROS generation induced by UVB or *t*-BHP in HaCaT cells (Figure 4B,C). Moreover, the antioxidant efficacy of CSL3 could protect HaCaT cells, and CSL3 restored cell viability which was decreased by UVB irradiation (Figure 4D). Cell death is roughly classified into programmed and unprogrammed cell death. Apoptosis is a representative example of programmed cell death, and ROS can induce apoptosis [39]. ROS decreases anti-apoptotic factors, such as the Bcl-2 family, but increases apoptosis-activating factors, such as Bax and PARP-1. PARP-1 is a well-known cellular substrate of caspases. Cleavage of PARP-1 by caspases is regarded as a hallmark of apoptosis [40]. CSL3 reduced the expression of cleaved caspase-3 and PARP-1. Additionally, Bax expression was also reduced by CSL3 treatment (Figure 4F). Thus, we concluded that CSL3 reduces UVB-induced ROS formation and protects HaCaT cells through inhibition of the apoptosis pathway.

ER stress can induce inflammation, aging, and even cancer in the skin [41,42]. ER stress is a mechanism to protect cells against various stimuli such as UPR, oxidative stress, and UVB irradiation [43]. However, if the ER stress persists, cell death, such as apoptosis, can be induced. Excessive UPR is a starting point for ER stress, which activates GRP78, phosphorylates IRE-1 and PERK, and later activates CHOP [44]. Therefore, we investigated whether CSL3 can suppress ER stress induced by UVB irradiation. We found that pretreatment with CSL3 inhibited ER stress caused by UVB irradiation (Figure 5A,B). Our results suggested that CSL3 can reduce skin aging by suppressing ER stress. However, further research is needed to identify a more concise molecular mechanism underlying CSL3-mediated ER stress inhibition.

Autophagy is important for maintaining cellular homeostasis by recycling damaged cellular organelles [3]. Accumulation of LC3B and reduction of p62 are representative features of autophagy. Moreover, mTOR inhibits autophagy by interfering with the formation of autophagosomes [45,46]. Our results showed that CSL3 activated autophagy through mTOR inhibition (Figure 6B) and activation of p-Akt, a well-known agent upstream of mTOR (Figure 6C). Thereafter, we investigated whether ER stress inhibition by CSL3 treatment was through autophagy regulation. 3-Methyladenine, an autophagy inhibitor, increased ER stress markers that were decreased by UVB irradiation (Figure 5D), whereas rapamycin, an autophagy inducer, reduced ER stress induced by UVB irradiation (Figure 5C). Because MAPKs are crucial regulators of mTOR and are involved in the suppression of ER stress [20], we inferred that the CSL3-mediated protective effect against UVB irradiation might be due to ERK-mTOR signaling. Additionally, Akt activity is affected by ERK phosphorylation [47,48], which may affect the UVB-mediated Akt-mTOR pathway [49]. Further studies are still required to validate the exact molecular mechanisms of the CSL3 effect after UVB irradiation; our group is currently investigating these mechanisms.

**Figure 9:**
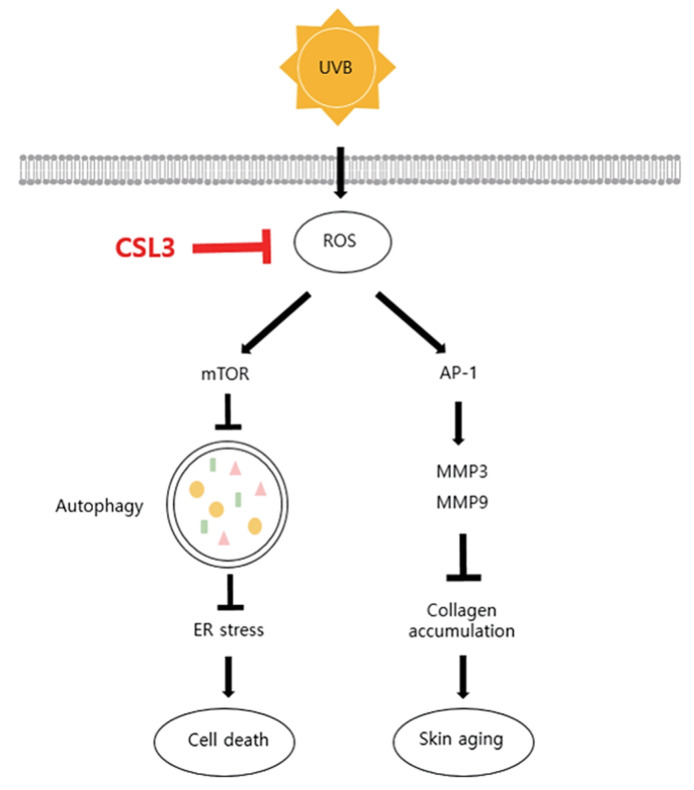
Schematic diagram illustrating the mechanism of CSL3 that protects against skin aging induced by UVB.

EGCG is contained at high levels in green tea and is known to have antioxidant properties [27,50]. We found that EGCG is a major constituent of CSL3, whose EGCG contents are much higher than in green tea (Figure 3B). EGCG is known to have a beneficial effect on UVB-damaged keratinocytes by preventing ROS generation [51] and suppressing various skin damages through its antioxidant capacity [52]. Our results confirmed that increased ROS generation after UV irradiation or *t*-BHP treatment is significantly reduced by EGCG treatment (Figure 8C). Although it was reported that EGCG could inhibit UVB-induced skin damage, its cellular and molecular mechanisms were unclear. We found that EGCG might regulate autophagy and ER stress in UVB-irradiated keratinocytes. We confirmed that EGCG induced autophagy to suppress ER stress, which can lead to improved cell viability, similar to CSL3 (Figure 8D). The second highest component in CSL3 after EGCG was rutin (Figure 3B). Rutin also exerts a cytoprotective effect on UV-damaged skin fibroblasts by decreasing ROS levels [53]. Therefore, these results suggested that the effect of CSL3 in UVB-irradiated keratinocyte damage may be due to various polyphenol components in CSL3, including EGCG and rutin (Figure 9).

It is still necessary to further investigate the cytoprotective effects and its mode of action of CSL3 in other keratinocytes, including primary cells and human N/TERT keratinocytes, as well as skin fibroblasts. Moreover, in vivo experiments are also required for developing CSL3 as a complementary candidate against UVB-induced skin damage. Although these limitations should be further overcome to consolidate and expand present findings, the present study concludes that CSL3 may be a promising candidate against UVB-induced photodamage.

## 4. Materials and Methods

### 4.1. Materials

Anti-LC3B antibody was purchased from NOVUS Biologicals (Littleton, CO, USA); anti-p62 antibody from Abnova (Taipei, Taiwan); anti-GRP78 antibody, from Abcam (Cambridge, MA, USA); anti-CHOP, phospho-ERK, ERK, phospho-JNK, JNK, phospho-p38, p38, phospho-mTOR, caspase-3, phospho-Akt antibodies from Cell Signaling Technology (Danvers, MA, USA) Bax and PARP antibodies were from Santa Cruz Biotechnology (Santa Cruz, CA, USA) and horseradish peroxidase-conjugated goat anti-mouse and anti-rabbit antibodies from Invitrogen (Carlsbad, CA, USA). Furthermore, 3-(4,5-dimethyl-2-thiazolyl)-2,5-diphenyl-2H-tetrazolium bromide (MTT), 2′,7′-dichlorofluorescein diacetate (DCFH-DA), *tert*-butyl hydroperoxide (*t*-BHP), epigallocatechin-gallate (EGCG), 2,2-diphenyl-1-picrylhydrazyl (DPPH), 2,2’-azino-bis(3-ethylbenzothiazoline-6-sulfonic acid) diammonium salt (ABTS), ethanol (EtOH), methanol (MeOH), sodium carbonate, aluminum chloride (AlCl_3_), Folin–Ciocalteu (F-C)’s phenol reagent, acetic acid, gallic acid, epicatechin, rutin, tannic acid, naringin, quercetin, and β-actin antibody were acquired from Sigma-Aldrich (St. Louis, MO, USA).

### 4.2. Chemical Extracts of CSL

Fresh CS leaves were collected from Wando-gun, Jeollanam-do, Korea, during the summer season of 2020 and dried at 40 °C. The dried leaves (10 g) were ground and extracted using several methods described in Figure 1. CSL powder was extracted with water by autoclaving at 1.0 atm and 100 °C for 1 h and using an ultrasonic bath (POWER SONIC 520, Hwashin Tech Co., Ltd., Seoul, Korea) at 25 °C for 4 h in another method. In the third method, conducted for 7 days at 25 ± 2 °C, 70% and 100% EtOH concentrations were used to extract CSL. The four extracts were then vacuum-concentrated. The final methods and solvents were named autoclaving (CSL1), ultrasonic (CSL2), 70% EtOH (CSL3), and 100% EtOH (CSL4).

### 4.3. DPPH Radical Scavenging Activity

DPPH radical scavenging activity was measured by Blois’s method [54] with slight modifications. Briefly, 200 μL of each concentration and 800 μL of 0.5 mM DPPH reagent were mixed and vortexed, followed by reaction in the dark for 15 min. Optical absorbance was measured at a wavelength of 517 nm using a Synergy HT multi-detection microplate reader (Biotek, Winooski, VT, USA). This process was repeated three times for each sample to obtain an average value, and ascorbic acid was used as a positive control. The radical scavenging activity of each solution was calculated using the following equation and expressed as a percentage: radical scavenging activity (%) = (Abs_control_ − Abs_sample_)/Abs_control_ × 100, where Abs_control_ is the absorbance of the MeOH control, and Abs_sample_ is the absorbance of the CSL extracts.

### 4.4. ABTS Radical Scavenging Activity

ABTS radical scavenging activity was measured using a method described by Re et al. [55] with slight modifications. Briefly, equal volumes of 7 mM ABTS and 2.45 mM potassium persulfate were mixed and reacted in the dark for 18 h to form ABTS radicals. The ABTS radical solution was diluted with distilled water, and the optical absorbance value at 730 nm was adjusted to 0.90 ± 0.02. Then, 200 μL of each sample concentration and 1000 μL of ABTS radical solution were mixed and vortexed, followed by reaction in the dark for 15 min. Absorbance was measured at a wavelength of 730 nm using the Synergy HT multi-detection microplate reader (Biotek). The radical scavenging activity of each solution was calculated using the following equation and expressed as a percentage: radical scavenging activity (%) = (Abs_control_ − Abs_sample_)/Abs_control_ × 100, where Abs_control_ is the absorbance of the MeOH control, and Abs _sample_ is the absorbance of the CSL extracts.

### 4.5. TPC and TFC

TPC (Total flavonoid contents) was measured using a modified Folin–Ciocalteu method [56]. Briefly, 500 μL of 1 mg/mL CSL extract, 500 μL of 0.2 M Folin–Ciocalteu’s phenol reagent (Sigma-Aldrich, USA), and 500 μL of 2% sodium carbonate were mixed and vortexed, followed by reaction in the dark for 30 min. Following a 30 min reaction, absorbance was measured at 750 nm using a Biotek Synergy HT multi-detection microplate reader. The measured value was converted into the amount of gallic acid (GAE) contained per 1 g of the CSL extract by substituting it into a standard calibration curve to obtain the total polyphenol content. TPC was expressed as mg/g of GAE based on a calibration curve with the following equation: y = 8.4755x + 0.1105, R^2^ = 0.9779, where x is the absorbance and y is the GAE (mg/g).

TFC (Total polyphenol contents) was measured by modifying the method described by Kim et al. [57]. After taking 500 μL each of a CSL extract of 1 mg/mL concentration and diluted quercetin standard solution for each concentration, 1.5 mL of methanol, 100 μL of 10% aluminum chloride, 100 μL of 1 M potassium acetate, and 2.8 mL of distilled water were added in that order to 40 μL at room temperature and reacted in the dark for 40 min. Absorbance was then measured at 415 nm using a Biotek Synergy HT multi-detection microplate reader. The measured value was converted into the amount of quercetin (QUE) contained per 1 g of the CSL extract by substituting it into a standard calibration curve to obtain the total flavonoid content. TFC was expressed as mg/g of quercetin equivalent based on a calibration curve with the following equation: y = 3.1736x + 0.0397, R^2^ = 0.9998, where x is the absorbance and y is the quercetin equivalent (mg/g).

### 4.6. Quantitative Analysis of Polyphenols Using HPLC MS/MS

The LC-MS/MS analysis was performed using an AB SCIEX 4000 Q Trap LC/MS/MS System (Shimadzu LC 20A System, Kyoto, Japan), and water (in 0.1% formic acid, solvent A) was used as the mobile phase in the analysis conditions, whereas acetonitrile (in 0.1% formic acid, solvent B) was used under isocratic conditions (35% B). Using Turbo Ion Spray, the analytical conditions of MS/MS were examined in both negative and positive modes.

### 4.7. HPLC with Diode-Array Detection (HPLC–DAD) Analysis

The CSL extracts (CSL1-CSL4) were quantitatively analyzed using HPLC-DAD (SPD-20A, Shimadzu Co., Japan). Seven standards (gallic acid, epicatechin, EGCG, rutin, tannic acid, naringin, and quercetin) were selected for experiments, and HPLC analysis conditions were as follows: Shim-pack GIS-ODS column (C18, 4.6 × 250 mm, 5.0 μm, Shimadzu Co.), flow rate of 0.7 mL/min, temperature 30 °C, injection volume 20 μL, and a UV detector wavelength of 280 nm. For the mobile phase, 0.1% acetic acid in water (solvent A) and 0.1% acetic acid in methanol (solvent B) were used. The gradient conditions of the mobile phase were 0 min: B (10%), 0–5 min: B (10%), 5–15 min: B (40%), 15–45 min: B (60%), 45–55 min: B (80%), 55–60 min: B (100%), 60–65 min: B (10%), 65–70 min: B (10%). The injection volume was 20 μL. All samples used for analysis were filtered with a 0.45 µm filter.

### 4.8. Cell Culture

HaCaT cells (human keratinocyte cell line) were cultured in 60 mm plates (Thermo Scientific, Waltham, MA, USA) and grown to 70–80% confluency. Cells were maintained in Dulbecco’s modified Eagle’s medium with 10% fetal bovine serum (Atlas Biologicals, Fort Collins, CO, USA) and 100 units/mL penicillin and 100 μg/mL streptomycin at 37 °C under a humidified 5% CO_2_ atmosphere. CSL3 was dissolved in DMSO at 1000 × stock concentration, and stock solution was diluted in culture medium for the treatment. EGCG was prepared in sterilized water.

### 4.9. MTT Assay

To measure the cytotoxicity of CSL3, HaCaT cells were plated at a density of 1 × 10^5^ cells/well in 24-well plates and treated with various concentrations of CSL3 for 12 h. Then, to identify the cytoprotective effect of CSL3 on photodamaged HaCaT cells, the cells were treated with CSL3 (30 or 100 μg/mL) for 1 h and incubated with UVB (30 mJ/cm^2^) or *t*-BHP (600 μM) for 6 h. After treatment, the cells were stained with MTT (0.2 mg/mL) for 30 min to identify viable cells. Formazan crystals produced in the wells were dissolved by adding 300 μL of dimethyl sulfoxide. The optical absorbance was measured at 550 nm using a microplate reader (Spectra MAX; Molecular Devices, Sunnyvale, CA, USA).

### 4.10. UVB Irradiation

HaCaT cells were seeded at 4 × 10^5^ cells/well in a 60 mm plate. After 24 h, the culture medium was replaced with serum-free medium, and the cells were incubated for 3 h. Then, the cells were treated with CSL3 (30 or 100 μg/mL) for 1 h. Then, the cells were washed with phosphate-buffered saline (PBS), followed by the addition of 1 mL PBS. Next, the cells were irradiated with UVB (source: UVB mercury lamp; range: 280–315 nm; dose 30 mJ/cm^2^) using a Bio-Link Crosslinker (Vilber Lourmat, Torcy, France). Following UVB irradiation, the cells were washed with PBS and cultured in a fresh medium.

### 4.11. Immunoblot Analysis

Protein extraction, SDS-polyacrylamide gel electrophoresis (SDS-PAGE), and immunoblot analysis were performed as previously reported [58]. Briefly, cell lysates were separated by SDS-PAGE (6%, 7.5%, and 12% acrylamide gels) and electrophoretically transferred to nitrocellulose (NC) membranes (GE Healthcare, Chicago, IL, USA). Subsequently, the NC membranes were blocked with 5% skim milk at 37 °C and incubated overnight at 4 °C with a primary antibody. After eliminating the primary antibody, the NC membranes were washed with PBS three times for 10 min. The NC membranes were incubated with a secondary antibody (Invitrogen) for 1 h at room temperature. After washing, membranes were treated with an enhanced chemiluminescence detection kit (Amersham Biosciences, Buckinghamshire, UK). Immunoreactive proteins were visualized using LAS 4000 (Fujifilm, Tokyo, Japan). β-actin was used as an immunoblotting control.

### 4.12. RNA Isolation and RT-PCR Analysis

TRIzol (Invitrogen) was used to obtain total RNA extracts according to the manufacturer’s protocol. To synthesize cDNA, total RNA (2 μg) was reverse-transcribed using an oligo-dT_18_ primer. The synthesized cDNA was amplified using a high-capacity cDNA synthesis kit (Bioneer, Daejeon, Korea) with a thermal cycler (Bio-Rad, Hercules, CA, USA). PCR-amplified products were separated by 2% agarose gel electrophoresis and staining with ethidium bromide (Sigma, St. Louis) and imaged using a gel documentation system (Fujifilm). The following PCR primer sequences were used: human MMP3 sense 5′-AACCTGTCCCTCCAGAACCT-3′ and antisense 5′-GGAAGAGATGGCCAAAATGA-3′; human MMP9 sense 5′-CTCGAACTTTGACAGCGACA-3′ and antisense 5′-GCCATTCACGTCGTCCTTAT-3′; human Col1A1 sense 5′-CACAGAGGTTTCAGTGGTTTGG-3′ and antisense 5′-GCACCAGTAGCACCATCATTTC-3′; human GADD153/CHOP sense 5′-AGGGAGAACCAGGAAACGGAAACA-3′ and antisense 5′-TCCTGCTTGAGCCGTTCATTCTCT-3′; human and human GAPDH sense 5′-GAAGGTGAAGGTCGGAGTC-3′ and antisense 5′-GAAGATGGTGATGGGATTTC-3′. *GAPDH* was used as a reference gene for normalization.

### 4.13. Measurement of ROS Production

DCFH-DA fluorescent probe was used to measure intracellular ROS production in cells pretreated with CSL3 (30 or 100 μg/mL) for 1 h and irradiated with UVB (30 mJ/cm^2^). After treatment, the cells were stained with 10 µM DCFH-DA for 30 min at 37 °C in the dark and then washed with PBS and harvested. The production of intracellular ROS was observed under a fluorescence microplate reader (Gemini; Molecular Devices) at excitation/emission wavelengths of 485 nm/530 nm, respectively.

### 4.14. Cell Apoptosis Assay

Cell apoptosis assays were performed using the FITC Annexin V Apoptosis Detection Kit (BD Biosciences, NJ, USA), following the manufacturer’s instructions and as previously described [59]. Briefly, HaCaT cells were transferred to serum-free medium for 3 h and treated with CSL3 (30 μg/mL) for 1 h. After treatment, the cells were irradiated with UVB (30 mJ/cm^2^) and then incubated for 3 h. Subsequently, the cells were washed with PBS three times, harvested using trypsin, and centrifuged (4 °C, 3000× *g*, 3 min). Pellets were resuspended in 1X Annexin V binding buffer and stained with 1% Propidium Iodide Staining Solution and 1% FITC Annexin V for 20 min in the dark. Apoptotic cells were measured using flow cytometry (Beckman-Coulter, Brea, CA, USA).

### 4.15. Procollagen Type I Measurement

The synthesized procollagen levels in culture media were assessed using Procollagen Type I C-peptide EIA Kit (Takara Biochemical Inc., Kusatsu, Japan) according to the manufacturer’s instructions. Briefly, HaCaT cells were seeded at 4 × 10^5^ cells/well in a 60 mm plate. Cells were irradiated with UVB (30 mJ/cm^2^) in the presence or absence of CSL3 (30 or 100 μg/mL). After treatment, the culture media was collected from the plate. Antibody-POD conjugate solution and sample were added to an antibody-coated microtiter plate and incubated at 37 °C for 3 h. Subsequently, the plate was washed with PBS four times, and substrate solution was added for 15 min. Optical absorbance was measured at 450 nm using a microplate reader (Spectra MAX, Molecular Devices) after adding stop solution.

### 4.16. Wound Healing Assay

A culture inserts kit (Ibidi, Gräfelfing, Germany) was used to evaluate cell migration. HaCaT cells were seeded at 5 × 10^5^ cells/well in culture inserts. The cells were irradiated with UVB (30 mJ/cm^2^) in the presence or absence of CSL3 (30 or 100 μg/mL). Then, culture inserts were removed. After 12 h, the scratched area was visualized in a microscope (Axiovert 200 M; Carl ZEISS, Jena, Germany).

### 4.17. Statistical Analysis

One-way ANOVA was used to assess significant differences between treatment groups. The Newman–Keuls test was used to assess significant differences between the means of multiple groups. Results are expressed as means ± standard error (SE).

## 5. Conclusions

Conclusively, our result showed that the 70% ethanol (EtOH) extract of *Castanopsis sieboldii* leaf (CSL3) has higher antioxidative capacity compared with the other CSL extracts. UVB irradiation leads to skin damage by inducing ROS and apoptosis. However, CSL3 succeeded in preventing UVB-induced cytotoxicity by suppressing ER stress through autophagy induction, resulting in increased cell viability. Additionally, CSL3 pretreatment increased collagen accumulation and cell migration, which were attenuated by UVB irradiation. Moreover, we found that EGCG is the major constituent of CSL3, and CSL3’s effects on keratinocytes were due to its antioxidant capability. Therefore, we propose that CSL3 may represent a promising candidate for the treatment of UVB-induced skin photodamage and skin aging.

## Data Availability

The data presented in this study are available in the article.

## Supplementary Materials

The following supporting information can be downloaded at: https://www.mdpi.com/article/10.3390/molecules28062842/s1, Figure S1: Identification of 15 polyphenols and quantitatively analyzed them in CSL1-4 extract by LC-MS/MS system. Figure S2: Determination of LC3II and CHOP expression by CSL3 alone. Figure S3: Determination of UVB ray absorption of CSL3.
