# Supplementary material for: Protective Effect of Castanopsis sieboldii Extract against UVB-Induced Photodamage in Keratinocytes
Source: Molecules. 2023 Mar 21;28(6):2842. doi: 10.3390/molecules28062842 (PMC10054760; doi:10.3390/molecules28062842)
Supplement: Supplementary file 1 [file molecules-28-02842-s001.zip › molecules-2225142-supplementary.pdf]

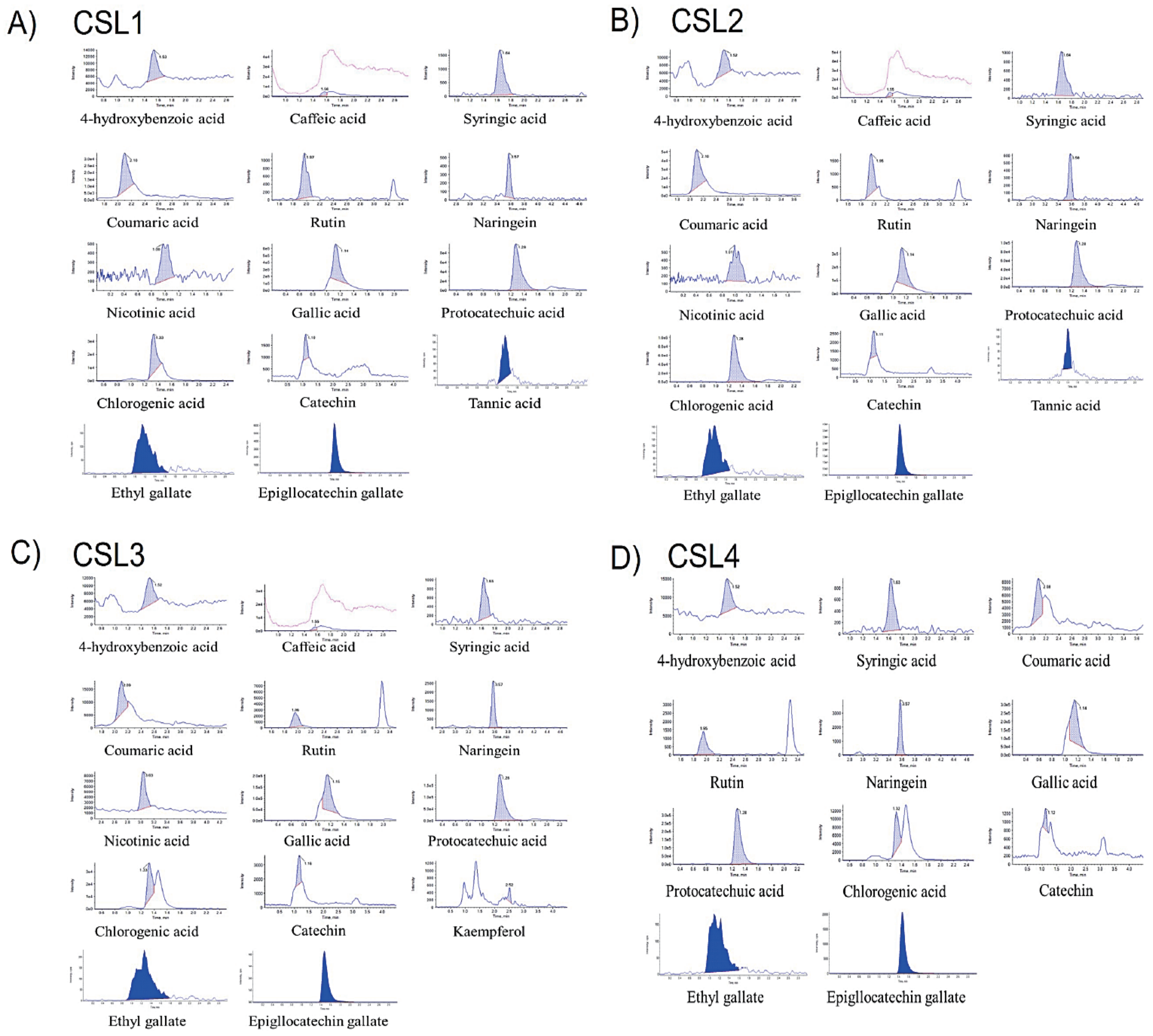

E)

No.	Standard	Calculated Concentration (ug/g)			
		CSL1	CSL2	CSL3	CSL4
1	4-hydroxy benzoic acid	17.14	13.3	15.55	17.94
2	Caffeic acid	50.46	50.11	25.73	-
3	Syringic acid	211.3	129.51	86.35	102.01
4	Coumaric acid	46.97	73.35	19.43	9.58
5	Rutin	14.15	16.06	28.24	18.95
6	Naringein	0.38	0.54	2.85	4.14
7	Nicotinic acid	5.72	6.52	9.45	-
8	Gallic acid	2248.67	1274.78	773.82	1252.83
9	Protocatechuic acid	271.26	274.41	497.03	816.21
10	Chlorogenic acid	496.51	523.94	448.35	134.5
11	Catechin	26.81	45.06	54.7	11.69
12	Kaempferol	-	-	7.3	-
13	Tannic acid	2.2	1.42	-	-
14	Ethyl gallate	18.6	14.8	25.4	19
15	Epigallocathechin gallate	25.1	170	2270	87.6
Total (ug/g)		3435.27	2593.8	4264.2	2474.45

Supporting Figure 1 (Lee et al.)

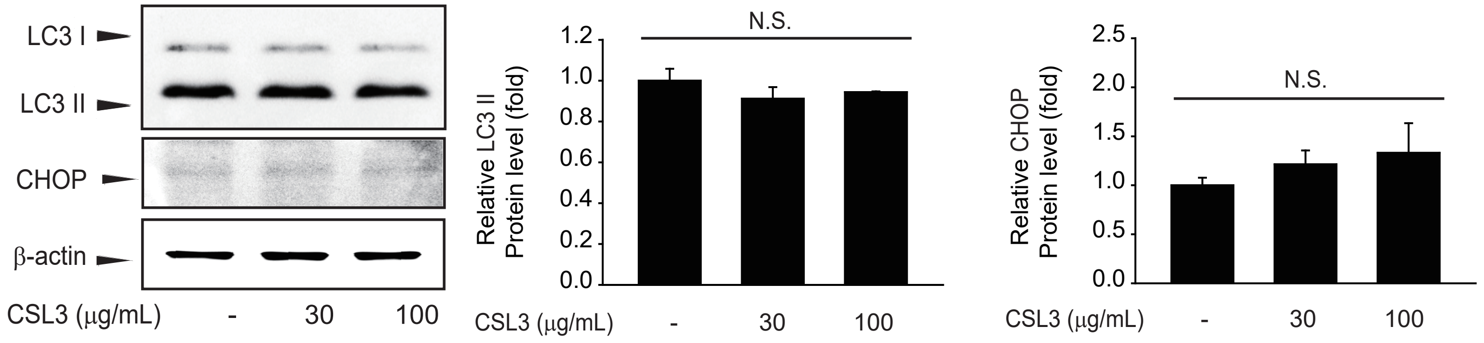

Supporting Figure 2 (Lee et al.)

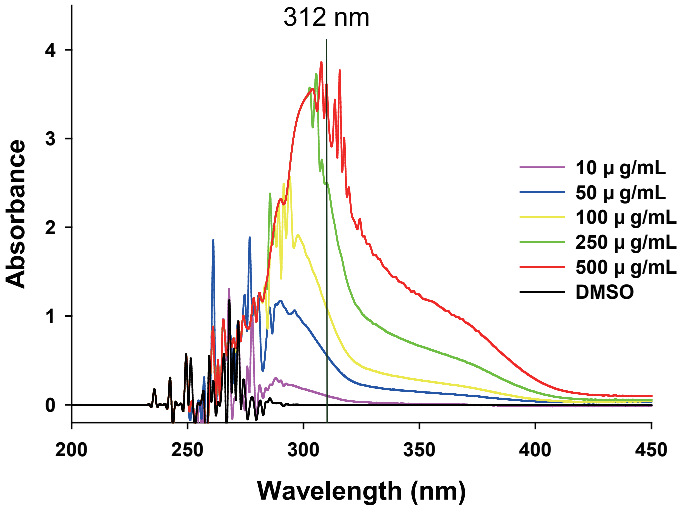

No.	Concentration	AU (312nm)
1	10 μg/mL	0.087 ± 0.002
2	50 μg/mL	0.487 ± 0.002
3	100 μg/mL	0.951 ± 0.001
4	250 μg/mL	2.215 ± 0.027
5	500 μg/mL	3.201 ± 0.285
6	DMSO	0.001 ± 0.007

Supporting Figure 3 (Lee et al.)
